# The positive mental health instrument: development and validation of a culturally relevant scale in a multi-ethnic asian population

**DOI:** 10.1186/1477-7525-9-92

**Published:** 2011-10-31

**Authors:** Janhavi Ajit Vaingankar, Mythily Subramaniam, Siow Ann Chong, Edimansyah Abdin, Maria Orlando Edelen, Louisa Picco, Yee Wei Lim, Mei Yen Phua, Boon Yiang Chua, Joseph YS Tee, Cathy Sherbourne

**Affiliations:** 1 Research Division, Institute of Mental Health/Woodbridge Hospital, 10, Buangkok View, 539747, Singapore; 2 RAND Corporation, Santa Monica, California, United Sates of America

**Keywords:** Positive mental health, multi-dimensional, instrument development, item reduction, factor analysis, item response theory

## Abstract

**Background:**

Instruments to measure mental health and well-being are largely developed and often used within Western populations and this compromises their validity in other cultures. A previous qualitative study in Singapore demonstrated the relevance of spiritual and religious practices to mental health, a dimension currently not included in exiting multi-dimensional measures. The objective of this study was to develop a self-administered measure that covers all key and culturally appropriate domains of mental health, which can be applied to compare levels of mental health across different age, gender and ethnic groups. We present the item reduction and validation of the Positive Mental Health (PMH) instrument in a community-based adult sample in Singapore.

**Methods:**

Surveys were conducted among adult (21-65 years) residents belonging to Chinese, Malay and Indian ethnicities. Exploratory and confirmatory factor analysis (EFA, CFA) were conducted and items were reduced using item response theory tests (IRT). The final version of the PMH instrument was tested for internal consistency and criterion validity. Items were tested for differential item functioning (DIF) to check if items functioned in the same way across all subgroups. **Results: **EFA and CFA identified six first-order factor structure (General coping, Personal growth and autonomy, Spirituality, Interpersonal skills, Emotional support, and Global affect) under one higher-order dimension of Positive Mental Health (RMSEA = 0.05, CFI = 0.96, TLI = 0.96). A 47-item self-administered multi-dimensional instrument with a six-point Likert response scale was constructed. The slope estimates and strength of the relation to the theta for all items in each six PMH subscales were high (range:1.39 to 5.69), suggesting good discrimination properties. The threshold estimates for the instrument ranged from -3.45 to 1.61 indicating that the instrument covers entire spectrums for the six dimensions. The instrument demonstrated high internal consistency and had significant and expected correlations with other well-being measures. Results confirmed absence of DIF.

**Conclusions:**

The PMH instrument is a reliable and valid instrument that can be used to measure and compare level of mental health across different age, gender and ethnic groups in Singapore.

## Background

Traditionally epidemiological studies have provided a wealth of research relating to the incidence, prevalence, determinants and consequences of mental illnesses, with little focus on mental health. The World Health Organisation states that health is a state of complete physical, mental and social well-being and not merely the absence of disease or infirmity and mental health is 'a state of well-being in which every individual realizes his or her own potential, can cope with the normal stresses of life, can work productively and fruitfully, and is able to make a contribution to her or his community' [1]. Mental health and well-being contribute to a wide range of outcomes for individuals and communities. These include the positive influence on lifestyle and behaviour [2], social performance [3], better quality of life [4], and fruitful ageing [5]. Given the positive outcomes of mental health and the growing realization of the serious limitations of relying solely on treatment and rehabilitation in mental illnesses, mental health promotion has emerged as a major health goal among policy makers. Although concerted efforts are being made worldwide to promote mental health in general, challenges exist in targeting efforts towards specific outcomes and measuring the effectiveness of such initiatives.

Singapore is a multi-ethnic country in Southeast Asia, with a population of 3.6 million citizens and permanent residents, of which 74.2% are of Chinese descent, 13.4% are of Malay descent, and 9.2% are of Indian descent [6]. Singapore has a high literacy rate (80.4%) and the main language of communication and commerce is English. In 2007, Singapore launched its First National Mental Health Policy and Blueprint and among its goals are the promotion of mental well-being and building resilience among its population with various initiatives planned to address these goals. While a number of instruments are available that measure mental health and well-being, most have been developed and used within the same population, and are unlikely to be valid in other countries as concepts of mental health may be unique and relevant to specific cultures [7-11] due to several reasons. Firstly, these instruments have been mainly developed and validated in Western populations and challenges with validity and appropriateness of adopting such measures across varied cultures have been reported [12,13]. Secondly the content of these measures relies either on literature, item reduction using item pool and expert panels [7,8,10,14], although it is generally recommended that the content of self-reported measures of well-being and quality of life be developed in the end-user [15,16]. In addition, most of the instruments either study a particular domain in greater detail using a lengthy questionnaire or are too short to provide meaningful comparisons and detection of change across different domains. Furthermore, very few measures are multi-dimensional, which is a well documented aspect of mental health [1] and hence crucial for its holistic assessment. Finally, in a preceding qualitative study conducted among adult participants belonging to the three major ethnic groups in Singapore, we identified the relevance of spiritual and religious practices to mental health in this population, a dimension which is largely neglected in the available multi-dimensional measures. In the qualitative study we conducted literature review to construct a framework of positive mental health followed by focus group discussions among adult participants belonging to the three major ethnic groups. The data from the study was used to generate an instrument with 182 candidate items.

The goal of this study was to develop the self-administered measure that covers all key and culturally appropriate domains of mental health, which can be applied to compare levels of mental health across different age, gender and ethnic groups. This study was conducted in two stages to further develop this instrument. The purpose of the first stage was to carry out item reduction while the second aimed to establish the validity of the measure in the local population. This paper describes the development of the instrument from factor analysis, item reduction and validation.

## Methods

### Ethics

Ethical approval was obtained from the Clinical Research Commiteee of the Institute of Mental Health and the Domain Specific Review Board of the National Healthcare Group, Singapore. Ethical approval covered all aspects of the study including design, sample size and selection, participant recruitment and data management procedures. A waiver of consent was obtained for the anonymous survey and return of completed questionnaires was considered as implied consent; the intent of the study and the details were conveyed to the participants using a study information sheet.

### Study design and participants

The study was conducted between April 2010 and February 2011. The details on time of assessments, sample size and analyses used in the two stages are depicted in Table 1. Singapore citizens or Permanent Residents (PRs) age 21-65 years, belonging to Chinese, Malay or Indian ethnicity, who were literate in English langauge were recruited through household purposive sampling, whereby only one respondent per household was permitted to participate, in order to avoid any bias. In addition, after targeting each household, interviewers were also instructed to skip two houses, before approaching the next household, to try and further reduce bias. Quota plans were developed to ensure an equal spread by age, gender and ethnicity and by geographic area, across Singapore. For the difficult-to-encounter cases (such as older PRs or English literate older residents) street intercepts at public areas such as malls, transport locations and community centres were carried out. Table 2 summarizes the socio-demographic characteristics of the participants from the two stages.

**Table 1:** Assessments, data collection and analyses of the two studies

	Stage 1	Stage 2
**Intent**	Item reduction	Validation

**Time line**	April 2010 - Sep 2010	Dec 2010 - Feb 2011

**Sociodemographic data** (age, gender, ethnicity, education, marital and employment status)	✓	✓

**PMH instrument**	182 candidate item scale, 4 point Likert style response scale (1- not at all like me, 2 - some what like me, 3 - moderately like me, 4- very much like me)	47-item scale, 6 point Likert style response scale (1- not at all like me, 2 - very slightly like me, 3 - slightly like me, 4- moderately like me, 5 - very much like me and 6- exactly like me)

**Other measures**	General Health questionnaire	RSA

	EQ5D	MSPSS

	General happiness item	Brief Cope

	General health item	PGIS

		DSES

		SWEMWBS

		SWLS

		General happiness item

		General health item

		EQ5D VAS

		Healthy days measure

		PHQ -8

		GAD -7

		SDS

**Analyses**	Missing data, floor and ceiling effect	Missing data, floor and ceiling effect

	EFA, CFA	CFA

	IRT-DIF	IRT-DIF

	Internal consistency	Internal consistency, Criterion validity

CFA: Confirmatory Factor Analysis; DSES:Daily Spirituality Experience Scale; EFA:Exploratory Factor Analysis;

EQ5D VAS: Euro-Quality of Life Scale Visual Analogue Scale; GAD-7:General Anxiety Disorder Scale;

IRT- DIF:Item response theory and Differential item functioning; MSPSS:Multi-dimensional Scale of Perceived Social Support; PGIS:Personal Growth Initiative Scale; PHQ-8:Patient Health Questionnaire; RSA:Resilience Scale for Adults

SDS: Sheehan Disability Scale; SWEMWBS:Short Warwick- Edinburg Mental Well-being Scale; SWLS: Satisfaction with Life Scale

**Table 2:** Demographic characteristics of the sample

		Stage 1 (N = 2088)		Stage 2 (N = 404)	
		**Mean**	**SD**	**Mean**	**SD**

**Age**		41.00	11.9	41.1	12.0

					

		**Freq**	**%**	**Freq**	**%**

**Gender**	Male	1036	49.62	197	49.0

	Female	1052	50.38	205	51.0

					

**Ethnicity**	Chinese	693	33.19	134	33.3

	Malay	699	33.48	123	30.6

	Indian	696	33.33	141	35.1

					

**Marital status**	Single	457	21.90	102	25.4

	Married	1486	71.20	288	71.6

	Separated/ Divorced/ Widowed	144	6.9	12	3.0

					

**Highest education attained**	Some formal education	8	0.38	3	0.8

	Primary	177	8.51	30	7.5

	Secondary	825	39.66	138	34.6

	Vocational	184	8.85	32	8.0

	'A' level	121	5.82	17	4.3

	Diploma	321	15.43	63	15.8

	Tertiary	444	21.35	116	29.1

					

**Current employment status**	Unemployed	517	24.76	138	34.2

	Employed	1569	75.14	266	65.8

Two major methodological changes were implemented between the two stages. These were:

1. The Positive Mental Health (PMH) instrument used in stage 1 comprised of a four-point response scale. However, some items were found to show ceiling effect and scoring required dichotomizing of the responses. To avoid compromising the responsiveness of the instrument, the four-point scale was expanded to a six-point scale following focus group discussions and cognitive testing.

2. To avoid any social desirability bias and counter possible floor/ceiling effect, during the second stage, interviewers issued the respondents a questionnaire along with a sealable envelope, instructing them to place the completed questionnaire in the envelope before collection. The questionnaires were kept with the respondent and not completed at the time of recruitment, as this method allowed respondents ample time to complete the questionnaire in privacy and reduced the likelihood of interviewer bias.

### Data collection

The information collected in the different stages included socio-demographic information about the participants, multiple questionnaires relating to domains of mental health and well-being and validity measures. The data collected in each stage are presented in Table 1 and included:

1. Socio-demographic information: age, gender, ethnicity, educational level, marital and employment status.

2. PMH instrument (Stage 1): The self-administered PMH instrument used in Stage 1 consisted of 182 candidate items and was developed through focus group discussions with 65 respondents in the three ethnic groups in a preceding study where five domains of PMH were deemed relevant to this multi-ethnic population. Briefly, the PMH items were developed to represent the following five domains: Personal growth and autonomy, relationships, spiritual beliefs and practices, Coping strategies and Personal characteristics. All PMH items were positively worded and respondents were asked to select a number showing how much the item described them on a four-point response scale, where '1' represented 'not at all like me' and '4' corresponded to 'very much like me'. Another domain on Global affect was added where respondents were asked to indicate 'how often over the past 4 weeks they felt - calm, peaceful, etc). The intention to add this domain was to be able to derive comparisons with the literature on 'Affect', which has been widely studied across multiple countries. 18 domain specific negatively worded filler items were also randomly distributed throughout the instrument. The purpose of including these items was to investigate pattern responses. These were subsequently not included in any analysis or scoring.

3. PMH instrument (Stage 2): Following factor analysis in Stage 1, the final instrument comprised 47 positively worded items representing the six domains of mental health. Respondents were presented with the statements along with a 6-item response scale for five domains (except for 'Global affect' domain). They were asked to select a number showing how much the item described them on the scale, where '1' represented 'not at all like me', '2' - very slightly like me', '3' - slightly like me, '4' - 'moderately like me', '5' - 'very much like me and '6' corresponded to 'exactly like me'. The 'Global affect' subscale included a list of five affect indicators and requires respondents to indicate 'how often over the past four weeks they felt - calm, peaceful, etc) using a 5-point response scale.

4. Validity measures: Fourteen validity measures were used to establish the criterion validity of the PMH instrument and its sub-domains. Measures were selected based on the similarity or divergence of the measure, based on expected and existing prior knowledge of their performance. Permission was obtained from the respective instrument developers or copyright holders before reproducing them in the questionnaires. The measures for convergent validity included a general happiness item, Satisfaction with Life Scale (SWLS) [17], two resilience measures - Brief COPE [18] and Resilience Scale for Adults (RSA) [19], Personal Growth Initiative Scale (PGIS) [20], Multi-dimensional Scale of Perceived Social Support (MSPSS) [21] and Daily Spirituality Experience Scale (DSES) [22]. Short Warwick-Edinburg Mental Well-being Scale (SWEMWBS) [23], and Euro-Quality of Life Scale (EQ5D) [24] were used as a global measures of mental health and health related quality of life we used the EQ5D Visual Analogue Scale (VAS) scores for the study. Divergent measures included the Generalised Anxiety Disorder (GAD)-7 Scale [25], Patient Health Questionnaire (PHQ)-8 [26], Sheehan Disability Scale (SDS) [27], general health item and Healthy Days Measure [28].

For the second stage, the socio-demographic questions, along with the PMH items and the subsequent validity measures were constructed into two separate questionnaires. All respondents received the socio-demographic questions, PMH items and the general health and happiness items, regardless of which version of the questionnaire they received. Due to the number of validity measures and their expected completion time, these measures were divided and split evenly between the two different versions of the questionnaire. Version one included the Healthy Days Measure, PHQ-8, EQ-5D, PGIS MSPSS and the SWLS. The second version of the questionnaire included the following validity measures; Brief COPE, GAD-7, SWEMWBS, SDS, DSES and the RSA. Both versions were similar in length, with regards to number of pages, estimated completion time and coverage of these measures. A brief description of the instruments is provided in Table 3.

**Table 3:** Brief description of validity measures used in the study

Instruments	N	Description
**Domains specific**		

RSA	201	This scale covers three main categories of resilience; dispositional attributes, family cohesion/warmth and external support systems, all of which contain various sub scales within each category. All items have an individual 5-point Likert scale which is specific to each individual item.

MSPSS	203	A 12-item self-report inventory that measures perceived social support from family, friends, and a significant other. Respondents use a 7-point Likert-type scale (very strongly disagree to very strongly agree) and scores are given for each of the three subscales as well as a total score.

Brief Cope	199	A 28-item self-report measure of both adaptive and maladaptive coping skills, consisting of 14 subscales, comprised of two items each. A 4-point Likert scale is used, whereby a higher score indicates greater coping strategies.

PGIS	201	Using a 6-point Likert scale from definitely disagree to definitely agree, this nine item, self-report instrument yields a single scale score for personal growth initiative (PGI), where a higher score indicates higher PGI.

DSES	172	A 16-item self-report measure designed to assess ordinary experiences of connection with the transcendent in daily life, which uses a modified 6-point Likert scale. Lower scores indicate less daily spirituality experience.

**Convergent measures**		

SWEMWBS	195	This 7-item uni-dimensional, self completed instrument measures positive mental well-being, where scores range from seven to 35 and higher scores indicate higher positive mental wellbeing.

SWLS	202	This 5-item instrument measures global cognitive judgments of satisfaction with one's life, using a 7-point scale from strongly disagree to strongly agree. Scores are summed and higher scores indicate higher satisfaction.

General happiness item	404	This single item asks respondents to rate their happiness, in general on a 7-point scale, where 1 = Not a very happy person and 7 = A very happy person.

General health item	404	This single item asks respondents to rate their health, in general on a 5-point scale from poor to excellent.

EQ5D VAS	190	A self-completed measure of health status comprising of a descriptive system which includes five dimensions (mobility, self-care, usual activities, pain/ discomfort and anxiety/ depression) and a visual self-rated health scale.

Healthy days measure	190	This instrument assesses perceived sense of well-being, via four items relating to 1) self-rated health, 2) physical health, 3) mental health and 4) limitations to usual activity due to physical or mental health, during the past 30 days. Respondents indicate the number of unhealthy days, where the maximum is 30 days.

**Divergent measures**		

PHQ -8	200	A self-administered depression scale that adopts a 4-point scale, where 0 = not at all and 3 = nearly everyday, respondents indicate how often they have been bothered by each of the items, in the past two weeks. Total scores range from 0 to 27, where scores of 20 and above indicate severe major depressive disorder.

GAD -7	190	A 7-item anxiety measure, where respondents are asked in the past two weeks how often they have been bothered by the following problems and use a 4-point scale from 'not at all' to 'nearly every day'. Scores are summed and higher score indicate greater anxiety.

SDS	201	A self report tool which assesses functional impairment via three inter-related domains; work/school, social and family life, using a 10-point visual analog scale. Scores are summed, whereby higher scores indicate higher impairment.

DSES:Daily Spirituality Experience Scale; EQ5D VAS: Euro-Quality of Life Scale Visual Analogue Scale; GAD-7:General Anxiety Disorder Scale; MSPSS: Multi-dimensional Scale of Perceived Social Support; PGIS:Personal Growth Initiative Scale; PHQ-8:Patient Health Questionnaire; RSA: Resilience Scale for Adults; SDS: Sheehan Disability Scale; SWEMWBS: Short Warwick- Edinburg Mental Well-being Scale; SWLS: Satisfaction with Life Scale

### Missing data and floor and ceiling effect

Missing data and floor and ceiling effect were investigated from frequency distributions of responses and were computed for each item, subscale and the overall PMH instrument. We also investigated if these differed by age, gender and ethnicity.

### Item reduction

This step was achieved in the first stage. Analyses were focused on item reduction through exploratory and confirmatory factor analysis, item response theory (IRT) and differential item functioning (DIF) [29], and correlations with other scales. Removal of the items was discussed with regard to both the statistical parameters and impact on the final instruments' content, taking into account the phrasing of the items and their meaning.

1. Exploratory factor analysis (EFA): The sample was randomly divided into two halves; one each for EFA and CFA. EFA for all 182 candidate items was implemented on the first random subsample (n = 1045) in order to determine the optimal factor solution for the item set and to identify poorly perfoming items for deletion. All factor analyses were conducted using MPLUS version 6.0 [30]. Criteria for number of factors included the number of eigenvalues greater than 1.0, ratio of first to second eigenvalue, pattern of loadings on each factor (i.e., number of non-loading or double-loading items), and interpretability of each solution. For item deletion, we considered item content, redundancy, loadings (loading < 0.40 on a single factor or loadings > 0.40 on more than one factor), and interpretability of factors[31].

2. Confirmatory factor analysis (CFA): After deleting poorly performing items and determining the best factor solution from the EFA, we conducted the CFA to determine the fit of the factor structure for the reduced set of items using polychoric correlations with weighted least squares with the mean- and variance-adjusted chi-square (WLSMV) estimator. Three criteria were used to indicate the goodness of fit of the hypothesized model: Comparative Fit Index (CFI) > 0.95 [32], Root Mean Square Error Approximation (RMSEA) ≤.06 [33] and Tucker-Lewis Index (TLI) > 0.90 [34]. Modification indices (MI) were explored in order to identify parameter misfit.

3. Item performance and final item reduction: We used IRT to examine the item properties within each factor and to identify any remaining items that may not be performing ideally. All IRT analyses were conducted using IRTPro Beta version [35]. The graded response model [36] was used to estimate item difficulty (the 'b' parameter) and item discrimination (the 'a' parameter) commonly referred to as the item slope, in each item. The item characteristic curves, item information and test information function curves were utilized for evaluating the performance of individual items within the scale. Additionally, we evaluated item fit with the S-X2 index [37,38]. Finally, we conducted DIF tests across ethnicity (Chinese, Malay, and Indian), gender and age groups (< 40 versus ≥ 40). This age cut-off was based on the mean age of the sample. Due to the number of comparisons within each DIF analysis, Benjamini-Hochberg false discovery rate adjustments were made to maintain a false discovery rate of .05 [39]. Identified DIF was examined closely for magnitude and potential influence and items displaying substantial DIF were considered for deletion.

### Scoring of the PMH instrument

For obtaining total PMH score, items were summed and divided by 47. Similarly the five subscale scores (those with 6-point response scale) were obtained by adding the chosen response options dividing by the respective number of items. The Global affect subscale was recoded into six level categories before scoring. Higher scores indicate greater perceived PMH.

### Validation

The final version of the shortened PMH instrument was tested for construct validity, DIF, reliability and criterion validity using data from the second stage.

1. CFA and IRT for the final instrument: CFA and IRT DIF analyses were similar to those used in the first stage. CFA was conducted in 404 participants using polychoric correlations. The model was further tested using CFA and IRT-DIF across ethnicity (Chinese, Malays, Indian), gender (male, female), and age (< 40 versus ≥ 40) by specifying the final model in seven distinct runs - one for each category.

2. Reliability and criterion validity: SAS software version 9.2 (SAS Institute, Cary, NC, USA) was used for these analyses. Internal consistency of each subscale was evaluated using Cronbach's alpha coefficient, in which the acceptable level was set at 0.7 [40]. The criterion validity was tested using Pearson correlation tests between the PMH instrument and the validity measure addressing different constructs of mental health, both positive and negative. Several hypotheses were set. For example, we hypothesized that the PMH subscale 'Personal growth and autonomy' would have a positive and high correlation with the PGIS and 'Emotional support' would have a positive and high correlation with all the MSPSS domains. In addition, we hypothesized that all components of the PMH instrument, including total score, would have positive and high to moderate correlations with SWEMWBS and EQ5D VAS. We expected an inverse relationship between the PMH instrument and scales that measure concepts related to mental illness or disability. For example, all components of PMH scale would have negative correlation with the GAD-7, SDS and PHQ-8 scales. All statistical significance was set at a p value of less than 0.05.

## Results

The socio-demographic distribution of the participants is presented in Table 2. The mean age of the participants was around 41. There were slightly more women than men. In the first stage, the missing data for the PMH instrument was in the range of 1.5% to 3.1%. None of the items demonstrated floor effect, however, ceiling effect was observed for 60% of the items with most (70%) respondents selecting the higher two response categories. For the second stage, missing data ranged from 0.2% to 2.5%. Some ceiling effect remained in about 15% of the items. For both the stages, missing data did not vary across subscales and the socio-demographic subgroups.

### Item reduction

EFA: The plot of eigenvalues for the 182 items indicated that four-, five-, and six-factor solutions were plausible. Upon examination of each of the rotated solutions, we concluded that the six-factor solution was optimal. This decision was based on the pattern of eigenvalues, the pattern of loadings and the interpretability of the solution. Using this six-factor solution, a total of 54 items were removed due to low loadings or multiple factor loadings. A further 49 items were eliminated from the item set because they contained redundant content and performed poorly relative to other items with similar content that were retained. Based on the content of the remaining items in each factor, we labeled them as follows: General coping, Personal growth and autonomy, Spirituality, Interpersonal skills, Emotional support, and Global affect.

CFA: We conducted CFA on the second random subsample (n = 1043) to test the fit of the 79 item, six-factor structure found in the EFA step. The results of goodness-of-fit indices indicated that a six-factor model did not fit the data well, based on cut off criteria for relative fit indices recommended by Hu and Bentler [32]. Although the TLI (0.98) value was high, the CFI (0.84) and RMSEA (0.07) indicated poor fit. To identify possible sources for this, we examined the model modification indices, and considered item loadings and content. Model improvements based on modification indices suggested the removal of 16 additional items. The CFA was rerun on the remaining 63 items, and the 6-factor model fit the data well (CFI = 0.96, TLI = 0.96, RMSEA = 0.04). Except for the relationship of Spirituality with Global affect (0.28), correlations among factors were high (ranging from 0.48 - 0.77), indicating that perhaps a second order factor model may be a more appropriate solution. Thus we estimated a final model that specified each of the six first-order factors loading on a higher-order factor labeled 'PMH'. This higher-order six-factor model provided excellent fit to the data (RMSEA = 0.04, CFI = .96, TLI = 0.96). The standardized loadings of the six-factors to the higher order factor were high and ranged from 0.55 to 0.90. The stages and reasons for deletion of items are illustrated in Table 4.

**Table 4:** Stages of item reduction from the initial 182 items

Analysis	Items removed	Reason (s) for removal	Items used for subsequent analysis
EFA	54	Poor factor loadings	128

	49	Redundant content, poor performance as compared to similarly worded items	79

CFA	16	Based on modification indices, item loading and content	63

Item performance	5	High ceiling effect	58

IRT-DIF	11	Demonstrated Dif across important subgroups	47

CFA: Confirmatory Factor Analysis; EFA:Exploratory Factor Analysis; IRT- DIF:Item response theory and Differential item functioning;

Item performance and final item reduction: The graded response model, showed poor fit at the item level, yielding extremely high and significant S - X^2 ^values indicating unacceptable fit for this model specification. This poor fit was likely due to the skewed response distributions for the majority of items (few respondents tended to endorse response options at the negative end of the scale). Thus we decided to modify this four-point response scale, and after evaluating different transformations, decided that a dichotomous scale resulting from collapsing categories 1-3 into a single category and leaving category 4 as is was optimal. The transformed items were recalibrated as dichotomous items and this specification provided acceptable results. We examined the item properties based on this set of calibrations and elected to remove five items from the Personal growth and autonomy factor because of low slope parameters.

Next we evaluated all items within each factor for DIF according to ethnicity, age (< 40 years and ≥ 40 years) and gender. Items were considered for deletion if they displayed DIF in large magnitude for at least one comparison, or displayed significant DIF across two or more comparisons. Based on these criteria, the following items were deleted: two items each from General coping, Personal growth and autonomy and the Emotional support factors (high magnitude DIF in ethnicity and gender DIF), two items from the Spirituality factor (high magnitude DIF in ethnicity and age), one item from the Interpersonal skills factor (high magnitude age DIF), and two items from the Global affect factor (high magnitude ethnicity DIF).

A final CFA estimation of the higher-order six-factor model using the remaining 47 items resulted in excellent fit (CFI = 0.98, TLI = 0.98, RMSEA = 0.03). The item loadings of the six factors were high and ranged from 0.65 to 0.95. The fit statistics of the higher-order six-factor model were also tested separately across the three ethnic groups and were found to fit reasonably well based on statistic indices across the groups (Chinese, CFI = 0.98, TLI = 0.98, RMSEA = 0.03; Malay, CFI = 0.98, TLI = 0.98, RMSEA = 0.03; and Indian, CFI = 0.98, TLI = 0.98, RMSEA = 0.03). Results from the final IRT calibrations for the reduced item set are shown in Table 5.

**Table 5:** Item parameter estimates (discriminant and difficulty) using 2-parameter logistic model for each six scales

Factor	Item No	a	**s.e**.	b	**s.e**.
F1. General coping					

	1.	2.32	0.13	0.39	0.04

	1.	2.57	0.15	-0.10	0.03

	1.	2.27	0.13	-0.01	0.03

	1.	2.40	0.14	0.52	0.04

	1.	2.19	0.13	0.23	0.03

	1.	2.45	0.14	0.06	0.03

	1.	1.93	0.11	0.64	0.04

	1.	2.31	0.13	0.18	0.03

	1.	2.33	0.13	0.04	0.03

					

F.2 Personal growth and autonomy	1.	3.16	0.18	0.21	0.03

	1.	3.03	0.17	0.26	0.03

	1.	2.73	0.15	0.50	0.04

	1.	2.87	0.16	0.09	0.04

	1.	2.85	0.16	0.25	0.03

	1.	3.30	0.20	-0.09	0.04

	1.	4.35	0.29	-0.02	0.03

	1.	2.88	0.17	0.20	0.03

	1.	3.81	0.26	0.15	0.03

	1.	2.88	0.17	0.28	0.03

					

F3. Spirituality	1.	2.32	0.13	0.17	0.04

	1.	3.49	0.22	0.32	0.03

	1.	4.34	0.30	0.19	0.03

	1.	3.17	0.19	-0.15	0.03

	1.	2.95	0.17	0.33	0.04

	1.	3.38	0.21	0.10	0.03

	1.	5.46	0.47	-0.06	0.03

					

F4. Interpersonal skills	1.	2.06	0.12	-0.05	0.04

	1.	1.98	0.11	-0.07	0.04

	1.	2.50	0.15	-0.02	0.03

	1.	2.71	0.16	0.01	0.03

	1.	2.51	0.15	0.27	0.04

	1.	2.54	0.15	0.21	0.03

	1.	1.85	0.11	0.03	0.04

	1.	2.32	0.14	0.23	0.04

	1.	2.56	0.15	0.15	0.03

					

F5. Emotional support	1.	1.21	0.08	0.43	0.05

	1.	1.12	0.07	-0.11	0.05

	1.	3.14	0.20	-0.15	0.03

	1.	2.25	0.14	-0.40	0.03

	1.	3.88	0.28	0.07	0.03

	1.	3.51	0.24	0.13	0.03

	1.	3.17	0.20	0.19	0.03

					

F6. Global affect	1.	2.78	0.19	0.89	0.04

	1.	3.60	0.27	0.46	0.03

	1.	4.17	0.35	0.47	0.03

	1.	3.21	0.23	0.69	0.03

	1.	2.09	0.13	0.78	0.04

					

Note. a represents the slope parameter estimates and b represents the difficulty parameter estimates

### PMH domain scores

The means and standard deviations of the PMH subscales and the overall scale scores, using the new scoring method, are presented in Table 6. The mean overall scale score among the participants was 4.3 (SD 0.7). There were significantly mild to moderate correlation coefficient (r = 0.25 to 0.70) between the six PMH subscales. The six subscales were strongly correlated with higher order PMH scale (correlation coefficient = 0.65 to 0.81).

**Table 6:** Mean, Standard Deviation of scores and Inter-correlations between PMH subscales

Variable	Mean	SD	Min	Max	Cronbach Apha		Positive Mental Health	General coping	Emotional support	Spirituality	Interpersonal Skill	Personal Growth & Autonomy	General Affect
													

**Positive Mental Health**	4.53	0.74	2	6		**Positive Mental Health**	1.00						

													

**General coping**	4.34	0.96	1	6	**0.89**	**General coping**	0.72*	1.00					

**Emotional support**	4.80	1.00	1	6	**0.89**	**Emotional support**	0.79*	0.48*	1.00				

**Spirituality**	4.29	1.49	1	6	**0.94**	**Spirituality**	0.65*	0.25*	0.35*	1.00			

**Interpersonal Skill**	4.69	0.84	2	6	**0.89**	**Interpersonal Skill**	0.79*	0.57*	0.66*	0.35*	1.00		

**Personal Growth & Autonomy**	4.64	0.88	2	6	**0.93**	**Personal Growth & Autonomy**	0.81*	0.61*	0.59*	0.29*	0.70*	1.00	

**General Affect**	4.37	0.98	1	6	**0.89**	**General Affect**	0.71*	0.47	0.49*	0.30*	0.45*	0.54*	1.00

* p value < 0.0001

### Validation

**Figure 1:**
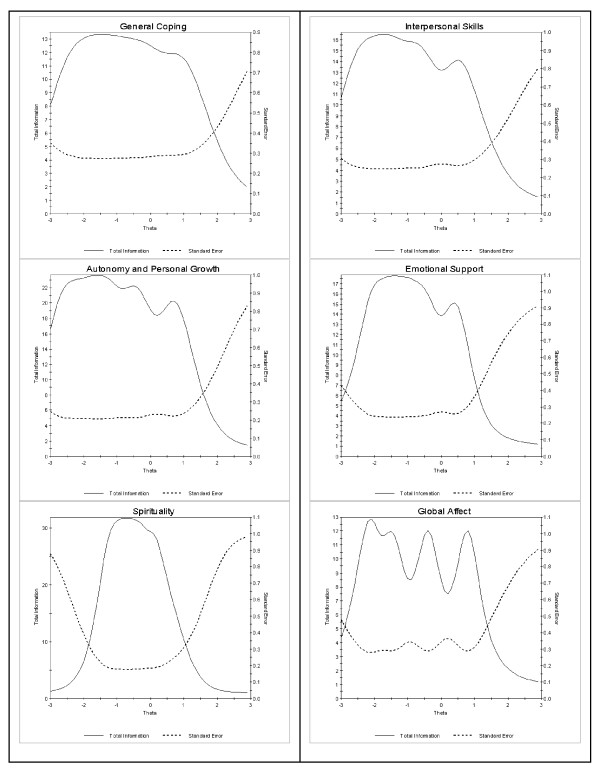
**Total Information Functions Curves for the Six Scales**.

CFA and IRT analyses: The CFA confirmed the higher-order six-factor model (RMSEA = 0.05, CFI = .96, TLI = 0.96). The standardized loadings of the six-factors to the higher order factor were high and ranged from 0.45 to 0.89 (Table 7). The results of goodness-of-fit indices fit the data well (CFI = 0.95-0.96, TLI = 0.95-0.96, RMSEA = 0.05-0.06) across ethnic, gender and age groups (Table 8). The slope estimates and strength of relation to the theta for all six PMH subscales were mostly high and acceptable. The slope estimates and strength of the relation to theta for all six PMH subscales were high and acceptable and ranged from 1.39 to 5.69 suggesting good discrimination properties. The threshold estimates for the instrument ranged from -3.45 to 1.61. Figure 1 displays six test information function curves for the 47 items from the six subscales. Test information function curves for all six subscales relatively peaked between -1.5 and - 1 on their underlying construct axis, which suggests that this scale provides higher precision at the lower end of the continuum (theta > 1). The standard error of measurement consequently increases in the high (theta > 1) range of theta. Among 47 items, some items displayed high magnitude of DIF including one General coping item, two spirituality items, and one Personal growth and autonomy item (Table 9). For example, within the 'General coping' subscale, we found the item "try not to take it too seriously" displayed higher than expected magnitude DIF between the younger and older age groups. Instead of removing the items we decided to keep these items due to their content and contribution to the construct.

**Table 7:** Factor loading of PMH instrument in overall sample and across gender, ethnicity and age groups

Factor	Item	Factor loading							
		**Total**	**Gender**	**Ethnicity**			**Age**		

			**Female**	**Male**	**Chinese**	**Malay**	**Indian**	**21-40y**	**41-65y**

		**N = 404**	**N = 205**	**N = 197**	**N = 134**	**N = 123**	**N = 141**	**N = 180**	**N = 184**

**General coping**	When I feel stressed ....								

	I try to move on	0.74	0.73	0.74	0.73	0.64	0.80	0.66	0.80

	I try not to let it bother me	0.76	0.73	0.80	0.70	0.72	0.81	0.65	0.85

	I tell myself that things would get better	0.78	0.75	0.80	0.83	0.66	0.75	0.74	0.80

	I try to relax	0.76	0.74	0.77	0.72	0.80	0.71	0.70	0.78

	I try not to take it too seriously	0.71	0.67	0.73	0.63	0.68	0.75	0.62	0.76

	I do something to get my mind off the situation	0.72	0.69	0.75	0.67	0.82	0.71	0.66	0.80

	I try to see it in a positive light	0.81	0.84	0.77	0.83	0.75	0.82	0.83	0.80

	I try to see the humorous side of the situation	0.63	0.68	0.58	0.61	0.60	0.64	0.60	0.66

	I try to solve the problem one step at a time	0.79	0.80	0.78	0.84	0.84	0.77	0.77	0.79

**Emotional support**	In general...								

	I spend time with people I like	0.76	0.78	0.71	0.74	0.71	0.82	0.79	0.73

	I try to get Emotional support from family and friends	0.54	0.61	0.49	0.51	0.65	0.48	0.49	0.55

	I have people in my life who give me support	0.84	0.83	0.86	0.84	0.85	0.84	0.82	0.81

	I have a close family	0.84	0.86	0.81	0.86	0.76	0.86	0.71	0.92

	When I have a problem there is someone I can go to for advice	0.83	0.84	0.83	0.88	0.74	0.81	0.84	0.81

	There is someone to cheer me up if I am having a bad day	0.86	0.84	0.88	0.91	0.73	0.94	0.88	0.83

	When I am in a difficult situation there is someone I can rely on	0.88	0.90	0.85	0.94	0.82	0.91	0.91	0.87

**Spirituality**	In general...								

	I find comfort in my religion or spiritual beliefs	0.85	0.84	0.87	0.86	0.82	0.75	0.87	0.87

	I believe God has a plan for me	0.88	0.86	0.89	0.91	0.78	0.78	0.90	0.88

	I set aside time for meditation or prayer	0.88	0.86	0.91	0.87	0.81	0.88	0.89	0.88

	I believe there is a higher being who looks after me	0.90	0.92	0.88	0.92	0.71	0.91	0.90	0.91

	I feel gods presence in my life	0.95	0.94	0.97	0.93	0.89	0.96	0.97	0.95

	I gain spiritual strength by trusting in a higher power.	0.84	0.82	0.85	0.93	0.61	0.81	0.86	0.80

	My religious beliefs influence the way I live	0.87	0.87	0.86	0.91	0.72	0.82	0.87	0.87

**Interpersonal skills**	In general...								

	I get along well with others	0.87	0.91	0.84	0.89	0.83	0.88	0.79	0.91

	I make friends easily	0.84	0.80	0.86	0.82	0.86	0.81	0.82	0.87

	I make an effort to help others	0.77	0.89	0.66	0.73	0.82	0.79	0.75	0.78

	I try to accept people as they are	0.77	0.80	0.75	0.78	0.65	0.85	0.71	0.83

	I am willing to compromise with people	0.66	0.68	0.65	0.61	0.66	0.68	0.61	0.76

	I try to be patient with others	0.74	0.75	0.73	0.75	0.65	0.78	0.68	0.80

	I am willing to give up something if it makes my family or friends happy	0.60	0.54	0.69	0.62	0.61	0.62	0.50	0.70

	I have no trouble keeping friends	0.76	0.73	0.80	0.75	0.76	0.77	0.75	0.80

	I am willing to share my time with others	0.75	0.79	0.71	0.76	0.77	0.75	0.75	0.81

**Personal growth and autonomy**	In general...								

	I have confidence in the decisions I make	0.84	0.85	0.84	0.87	0.79	0.85	0.82	0.86

	I feel comfortable expressing my opinions	0.81	0.80	0.83	0.80	0.85	0.78	0.81	0.83

	I am able to control many situations around me	0.81	0.75	0.87	0.84	0.79	0.79	0.83	0.79

	I have freedom to make choices that concern my future	0.78	0.77	0.80	0.85	0.75	0.76	0.73	0.84

	I feel in control of my life	0.60	0.49	0.69	0.69	0.45	0.66	0.61	0.60

	I work hard to achieve my goals	0.82	0.79	0.85	0.78	0.76	0.87	0.82	0.81

	I am clear about what I want in life	0.85	0.86	0.85	0.88	0.74	0.92	0.88	0.82

	I am able to solve my own problems	0.83	0.81	0.84	0.84	0.80	0.85	0.79	0.86

	I am focused on what I want to do in life	0.87	0.85	0.88	0.86	0.84	0.88	0.84	0.90

	I know what I need to do to reach my goals	0.84	0.83	0.84	0.81	0.80	0.88	0.83	0.88

**Global affect**									

	calm	0.77	0.82	0.71	0.67	0.82	0.84	0.73	0.75

	happy	0.88	0.91	0.85	0.87	0.84	0.92	0.87	0.90

	peaceful	0.92	0.91	0.93	0.89	0.90	0.98	0.96	0.88

	relaxed	0.84	0.88	0.80	0.80	0.87	0.83	0.83	0.87

	enthusiastic	0.75	0.76	0.74	0.83	0.67	0.71	0.70	0.78

**Table 8:** Correlations within PMH domains and Fit indices

	Total	Gender			Ethnicity			Age
		**Female**	**Male**	**Chinese**	**Malay**	**Indian**	**21-40y**	**41-65y**

General coping	0.75	0.72	0.78	0.78	0.66	0.80	0.73	0.75

Emotional support	0.80	0.78	0.85	0.78	0.80	0.79	0.82	0.81

Spirituality	0.45	0.41	0.50	0.19	0.62	0.50	0.50	0.45

Interpersonal skills	0.89	0.84	0.93	0.94	0.92	0.80	0.85	0.91

Personal growth and autonomy	0.88	0.88	0.90	0.89	0.92	0.85	0.84	0.92

Global affect	0.66	0.67	0.69	0.77	0.43	0.72	0.69	0.64

								

CFI	0.96	0.95	0.96	0.96	0.93	0.95	0.95	0.96

TLI	0.96	0.95	0.96	0.95	0.93	0.95	0.95	0.96

RMSEA	0.05	0.06	0.05	0.06	0.06	0.06	0.06	0.06

CFI- Comparative fit indexes, RMSEA- Root Mean Square Error Approximation, TLI - Tucker-Lewis Index

**Table 9:** Item parameter estimates (discriminant and difficulty) using Samejima Graded response (IRT) model for each six scales

Factor	Item	*a*	*s.e*.	*b*_1_	*s.e*.	*b*_2_	*s.e*.	*b*_3_	*s.e*.	*b*_4_	*s.e*.	*b*_5_	*s.e*.
**General coping**													

	1	1.94	0.17	-2.55	0.22	-1.79	0.15	-1.08	0.10	-0.12	0.08	1.09	0.11

	2	2.08	0.18	-2.34	0.19	-1.55	0.12	-0.89	0.09	0.05	0.08	1.42	0.13

	3	2.21	0.20	-2.55	0.21	-1.95	0.15	-1.23	0.10	-0.43	0.07	0.76	0.09

	4	2.35	0.21	-2.59	0.21	-1.79	0.13	-1.19	0.10	-0.31	0.07	0.82	0.09

	5	2.11	0.18	-2.25	0.18	-1.50	0.12	-0.72	0.08	0.08	0.08	1.32	0.12

	6	1.98	0.18	-2.55	0.22	-1.91	0.15	-1.10	0.10	-0.34	0.08	0.91	0.10

	7	2.62	0.23	-2.28	0.17	-1.61	0.12	-0.82	0.08	-0.05	0.07	0.98	0.09

	8	1.74	0.16	-1.75	0.15	-1.10	0.11	-0.37	0.08	0.48	0.09	1.61	0.15

	9	1.89	0.17	-2.85	0.26	-1.93	0.16	-1.20	0.11	-0.26	0.08	0.96	0.11

**Emotional support**													

	1	3.15	0.26	-2.62	0.21	-2.06	0.14	-1.39	0.10	-0.40	0.07	0.74	0.08

	2	2.42	0.20	-2.62	0.22	-2.22	0.17	-1.32	0.10	-0.40	0.07	1.00	0.10

	3	2.55	0.21	-2.31	0.18	-1.83	0.13	-0.99	0.09	-0.08	0.07	1.30	0.11

	4	2.38	0.20	-2.73	0.23	-2.11	0.16	-1.41	0.11	-0.53	0.07	0.77	0.09

	5	1.61	0.14	-2.47	0.22	-1.90	0.16	-1.10	0.11	-0.13	0.08	1.35	0.13

	6	2.73	0.23	-3.12	0.32	-2.22	0.16	-1.49	0.11	-0.62	0.07	0.48	0.08

	7	3.31	0.28	-2.57	0.20	-1.84	0.12	-1.29	0.09	-0.50	0.07	0.56	0.08

	8	2.89	0.24	-2.92	0.27	-2.17	0.16	-1.35	0.10	-0.40	0.07	0.86	0.09

	9	3.37	0.28	-2.55	0.20	-1.69	0.11	-1.11	0.08	-0.35	0.06	0.79	0.08

	10	3.00	0.25	-2.84	0.25	-1.99	0.14	-1.28	0.09	-0.35	0.07	0.79	0.08

**Spirituality**													

	1	2.56	0.22	-1.54	0.12	-1.13	0.09	-0.71	0.08	-0.34	0.07	0.39	0.08

	2	3.84	0.35	-1.29	0.09	-1.00	0.08	-0.63	0.07	-0.32	0.06	0.26	0.07

	3	3.03	0.25	-1.16	0.09	-0.71	0.08	-0.36	0.07	0.08	0.07	0.68	0.08

	4	3.66	0.32	-1.36	0.10	-1.05	0.08	-0.70	0.07	-0.26	0.06	0.36	0.07

	5	5.69	0.61	-1.22	0.08	-0.90	0.07	-0.62	0.06	-0.30	0.06	0.13	0.06

	6	3.25	0.27	-1.13	0.09	-0.75	0.08	-0.42	0.07	0.01	0.07	0.78	0.08

	7	3.46	0.29	-1.32	0.10	-0.84	0.08	-0.50	0.07	-0.06	0.07	0.65	0.08

**Interpersonal skills**													

	1	3.34	0.32	-2.54	0.21	-1.93	0.13	-1.46	0.10	-0.72	0.07	0.50	0.07

	2	3.03	0.28	-2.32	0.18	-1.64	0.12	-1.05	0.08	-0.44	0.07	0.49	0.07

	3	2.26	0.20	-3.01	0.29	-2.34	0.19	-1.69	0.13	-0.69	0.08	0.56	0.09

	4	2.14	0.19	-2.96	0.29	-2.38	0.20	-1.42	0.12	-0.43	0.08	0.88	0.10

	5	1.69	0.15	-3.04	0.30	-2.39	0.21	-1.32	0.12	-0.22	0.08	1.29	0.13

	6	1.98	0.18	-2.78	0.25	-2.02	0.16	-1.36	0.12	-0.37	0.08	1.06	0.11

	7	1.39	0.14	-3.45	0.38	-2.51	0.24	-1.59	0.16	-0.51	0.10	0.98	0.13

	8	2.56	0.23	-2.24	0.17	-1.77	0.13	-1.25	0.10	-0.62	0.07	0.74	0.09

	9	2.42	0.21	-2.71	0.24	-1.91	0.14	-1.33	0.10	-0.40	0.07	0.96	0.10

**Personal growth and autonomy**													

	1	3.15	0.26	-2.62	0.21	-2.06	0.14	-1.39	0.10	-0.40	0.07	0.74	0.08

	2	2.42	0.20	-2.62	0.22	-2.22	0.17	-1.32	0.10	-0.40	0.07	1.00	0.10

	3	2.55	0.21	-2.31	0.18	-1.83	0.13	-0.99	0.09	-0.08	0.07	1.30	0.11

	4	2.38	0.20	-2.73	0.23	-2.11	0.16	-1.41	0.11	-0.53	0.07	0.77	0.09

	5	1.61	0.14	-2.47	0.22	-1.90	0.16	-1.10	0.11	-0.13	0.08	1.35	0.13

	6	2.73	0.23	-3.12	0.32	-2.22	0.16	-1.49	0.11	-0.62	0.07	0.48	0.08

	7	3.31	0.28	-2.57	0.20	-1.84	0.12	-1.29	0.09	-0.50	0.07	0.56	0.08

	8	2.89	0.24	-2.92	0.27	-2.17	0.16	-1.35	0.10	-0.40	0.07	0.86	0.09

	9	3.37	0.28	-2.55	0.20	-1.69	0.11	-1.11	0.08	-0.35	0.06	0.79	0.08

	10	3.00	0.25	-2.84	0.25	-1.99	0.14	-1.28	0.09	-0.35	0.07	0.79	0.08

**Global affect**													

	1	2.75	0.23	-2.33	0.18	-1.7	0.12	-0.35	0.07	0.93	0.08		

	2	3.15	0.29	-2.44	0.2	-1.76	0.12	-0.59	0.07	0.68	0.07		

	3	4.32	0.45	-2.15	0.15	-1.43	0.09	-0.38	0.06	0.77	0.07		

	4	3.63	0.35	-2.33	0.18	-1.37	0.09	-0.17	0.06	0.91	0.08		

	5	1.82	0.16	-2.73	0.24	-1.48	0.13	-0.11	0.08	1.11	0.11		

Reliability: The Cronbach's alpha coefficient for the total score was 0.96. The alpha coefficients for General coping, Personal growth and autonomy, Spirituality, Interpersonal skills, Emotional supports and Global affect scores were 0.89, 0.93, 0.94, 0.89, 0.89, and 0.89 respectively.

Criterion Validity: Table 10 shows the Pearson correlation coefficient between the PMH instrument and other scales. All the six subscales of the PMH instrument and their total score (r ranged from 0.18 to 0.66, p value < 0.001) positively correlated with SWEMWBS. The spirituality subscale correlated highest, as expected, with the DSES spirituality scale (r = 0.76) and the correlation was weakest with the SWEMWBS. The correlation coefficients between all components of the PMH instrument and the SWLS ranged from 0.24 to 0.54 (p value < 0.01). The correlation coefficient between the PMH 'General coping' subscale and the Brief Cope Planning and Acceptance subdomains were 0.21 and 0.30 respectively. Our Personal growth and autonomy was positively and highly correlated with PGIS validity scale. The Global affect subscale showed highest and positive correlations with EQ5D VAS, SWEMWBS, general happiness and general health measures. As expected, the PMH instrument negatively correlated with the divergent scales that measured concepts related to mental illness and disability or impairment.

**Table 10:** Pearson correlations between the Positive Mental Health Scale and other established instrument

	General coping	Personal growth & autonomy	Interpersonal Skill	Spirituality	Emotional support	General affect	Positive Mental Health
RSA	0.32***	0.51***	0.49***	0.15*	0.43***	0.41***	0.49***

MSPSS							

MSPSS (Family)	0.37***	0.57***	0.56***	0.43***	0.66***	0.48***	0.69***

MSPSS (Friends)	0.29***	0.43***	0.53***	0.19**	0.62***	0.34***	0.54***

MSPSS (Other Subscale)	0.31***	0.53***	0.47***	0.34***	0.68***	0.48***	0.62***

MSPSS (Total Score)	0.37***	0.60***	0.59***	0.38***	0.75***	0.51***	0.71***

Brief Cope (BC) Scale							

BC (Planning Subscale)	0.21**	0.23**	0.20**	0.07	0.27**	0.08	0.22**

BC (Acceptance Subscale)	0.30***	0.30***	0.32***	0.26**	0.30***	0.32***	0.40***

BC (Humor Subscale)	0.11	0.16*	0.10	0.11	0.18**	0.15*	0.18**

PGIS	0.52***	0.72***	0.49***	0.30***	0.44***	0.51***	0.63***

DSES	0.11	0.12	0.24**	0.76***	0.13	0.28**	0.41***

SWEMWBS	0.53***	0.65***	0.51***	0.18**	0.48***	0.66***	0.66***

SWLS	0.44***	0.49***	0.41***	0.24**	0.43***	0.51***	0.54***

General happiness	0.41***	0.49***	0.39***	0.21***	0.41***	0.62***	0.58***

General health	0.32***	0.35***	0.27***	0.12*	0.22***	0.42***	0.36***

EQ5D VAS	0.36***	0.34***	0.25**	0.14*	0.25**	0.52***	0.39***

Healthy Days Measure	-0.33***	-0.33***	-0.36***	-0.14	-0.36***	-0.58***	-0.46***

PHQ-8	-0.35***	-0.36***	-0.28***	-0.20**	-0.30***	-0.61***	-0.46***

GAD-7	-0.14*	-0.29***	-0.08	-0.12	-0.12	-0.50***	-0.29**

Global Functioning From SDS	-0.15*	-0.12	-0.10	0.05	-0.08	-0.15*	-0.07

SDS							

SDS (Work/School)	-0.13	-0.22**	-0.13	-0.07	-0.16*	-0.36***	-0.23**

SDS (Social Life)	-0.17*	-0.26**	-0.15*	-0.14	-0.24**	-0.44***	-0.32***

SDS (Family life/Home responsibilities)	-0.07	-0.17*	-0.13	-0.10	-0.21**	-0.40***	-0.24**

SDS (Days Lost)	-0.13	-0.25**	-0.19*	-0.04	-0.26**	-0.34***	-0.20**

SDS (Days Unproductive)	-0.13	-0.28	-0.20**	-0.10	-0.27**	-0.41***	-0.27**

* p < 0.05; ** p < 0.01; *** p < 0.001

DSES:Daily Spirituality Experience Scale; EQ5D VAS: Euro-Quality of Life Scale Visual Analogue Scale; GAD-7:General Anxiety Disorder Scale; MSPSS: Multi-dimensional Scale of Perceived Social Support; PGIS: Personal Growth Initiative Scale; PHQ-8:Patient Health Questionnaire; RSA: Resilience Scale for Adults; SDS: Sheehan Disability Scale; SWEMWBS: Short Warwick- Edinburg Mental Well-being Scale; SWLS: Satisfaction with Life Scale

## Discussion

The applicability of existing instruments is marred by the lack of easily administrable, multi-dimensional instruments that cover all the culturally relevant domains of mental health. In this study, we demonstrated the validity and reliability of the PMH instrument using a series of studies in the local multiethnic population. Content of the PMH instrument was strengthened by identifying the components of the instrument through studies directly conducted among the end users. Though this method is now largely advocated for instrument content development, many of the available measures for well-being and patient reported outcomes have been developed by reducing item pools created from existing instruments [41,42], hence the content of our PMH measure encompasses experiences that are of relevance to the general population in Singapore.

Factor analysis uncovered six important dimensions of mental health in Singapore. Much attention was given to understanding the content in the factors before naming them. The assessment was theory-driven where we compared and contrasted the item content with the definitions of key domains from the extant well-being literature as well as looked at the content of the available measures. While reviewing the 'General coping' items, we observed a mixture of active coping and avoidance. The domain had items such as 'I try to see the looking at humorous side' and 'I tell myself that things would get better', which are not direct acts of coping, yet contribute to the process, hence we used the General coping instead of active or passive coping. Interpersonal skills, Emotional support, and Global affect were named based on the item structure and comparison with other definitions. There is an overlap of the theories on personal growth, autonomy and environmental mastery (EM), however, EM involves much more than just these two aspects [43]. The basis of EM is to be able to control situations surrounding the individual and turning the situation in favor of his/her needs. While we observed 'feeling in control' in the domain, the later was not evident. The content was also more comparable with definitions of autonomy and personal growth [20,43] and hence we labeled this domains as 'Personal growth and autonomy'.

Some of the dimensions are close to those reported in the literature, such as autonomy, personal growth, coping and support. While others such as interpersonal skills and spirituality emerged salient in the local population. These findings strongly justify our decision to develop a new measure directly in the local population instead of using existing measures. The role of spirituality in achieving PMH and particularly its interaction with other domains has been under explored, and as such, this instrument may be of interest to assess the socio-cultural aspects and influence on PMH.

In this study we conducted item reduction for the PMH instrument and demonstrated its factor structure using a series of psychometric analyses. While we used quota sampling strategy where we oversampled participants from the minority ethnic groups (Malay and Indian), weighted analysis was not conducted as the purpose of oversampling was to get enough power for statistical analyses on cultural difference tested in the CFA and IRT-DIF and these inferences could not be drawn using weighted analysis. We also did not devise weighted summation scores as we wanted to preserve the variation in the original data. Furthermore, they are not always applicable while applying the scale in populations other than the one they were derived from [44].

A key feature of this instrument remains the use of both classic and modern test theory practices to select items for inclusion in the PMH instrument. As the results showed, classic approaches to item reduction enabled the removal of most items, but failed to discriminate adequately between items with similar properties. This was addressed by the use of IRT which enabled us to examine the reasons for these issues in more detail. Another important aspect of this study is that we used IRT models that provide up to three parameters and allows for analysing response scales with multiple options [45]. In addition, these various approaches to item reduction ensured that the item correlations were substantially reduced and reflected construct validity by reduced redundancy.

The multi-dimensional PMH instrument has high internal reliability and fulfills assumptions with convergent and divergent validity. IRT thresholds (Table 5 theta < 0) and location estimates (peak in the negative zone) suggest superior accuracy in measuring mental health of individuals with below average levels of the domains General coping, Personal growth and autonomy, Spirituality, Interpersonal skills, and Emotional support. Global affect however, functions in the opposite direction as it will potentially provide more information on individuals above the average (theta > 0) and was slightly reduced when the theta was greater than 1.

A recent study of the SWEMWB indicated a multi-dimensional structure for the shortened well-being measure [23]. In an earlier study on the longer version of this scale (WEMWB), differing strengths of association with convergent validity variables were reported [7]. The correlations observed for our instrument were lower than those reported for WEMWBS. For example, WEMWBS demonstrated significantly strong correlation with EQ5D VAS (0.43) and SWLS (0.73) [7] where as the global PMH instrument measure showed a significant correlation of 0.39 and 0.53 with EQ5D VAS and SWLS, respectively. The differences in magnitude of correlations could be attributed to the study sample - WEMWBS estimates were established in a student population while ours were in a community sample, who were considerably older (mean age 41 y) and age is often associated with lower life satisfaction [46]. In our sample SWEMWBS score was significantly correlated (r = 0.380, p < 0.01) with SWLS. However, we couldn't investigate it's relation with EQ VAS as the questionnaires were administered in different groups of the sample.

The multi-dimensional PMH instrument developed in this study has several strengths. First, the conceptual framework of the instrument was based on qualitative and quantitative tests in the target population. The instrument includes six dimensions which encompass the notion that mental health can be achieved by the balance and strengths of multiple domains, and while an individual may not be equipped with all the components of mental health, an optimum level can be achieved through further strengthening the stronger components. Furthermore, the acceptability of the instrument is high (as evidenced by the low rates of missing data) across the different age, gender and ethnic groups for all domains.

While the PMH instrument demonstrated superior reliability and validity in the study population, some limitations of this study should be addressed. The study was limited to English speaking adults, aged 21 - 65 years. Therefore, other dimensions of mental health in the wider population such as the adolescents or the elderly may have fallen outside the scope of this study. Measures like EQ5D VAS and SWLS have been previously employed for validation of the WEMWBS [7], but most of the other converging validity measures were selected based on the expected performance. Nevertheless our study provides evidence that they can be reliably used to assess domains of PMH. We also observed significant but lower correlations with the shorter measures for some subscales. For example, association between the one-item general happiness measure and Spirituality were low (0.21, p < 0.01) while the two-item Brief Cope subscale scores were low across all domains. The use of few-item criterion measures may have constrained the strength of the associations between these criterion measures and the six separate factors of PMH. Although the length of the instrument (47 items) was not a limitation in our study, a shorter measure would be more appropriate in other settings and is planned as part of future research. We did not establish the test retest reliability of the measure. We were also unable to obtain information on attrition rates for the surveys as many participants were unwilling to provide basic background information upon declining participation.

## Conclusion

Based on our findings, we endorse the theory that mental health in adults is unlikely to be a one-dimensional construct. To fully understand the influence of the multiple domains of mental health, and to develop effective mental health promotion measures, all the relevant features of mental health need to be captured. The implications of having a culturally appropriate instrument to measure positive mental health are widespread and are essential in Singapore and ultimately will contribute to improved health outcomes in the population. The PMH instrument can be used to collect data on individuals and various sub groups in the population which would be crucial when reviewing existing mental health policy and services. Such information may also contribute to adequate mental health training, education and public awareness. An additional implication of using this instrument in a research setting will be to measure and observe changes to positive mental health among the Singapore population, over time. Further psychometric research is however, needed to establish the responsiveness, validity and reliability of the instrument in Singapore and other Asian and Western cultures.

## Competing interests

The authors declare that they have no competing interests.

## Authors' contributions

JAV led the study design, literature review, item reduction, field strategy for the survey and drafted the manuscript. MS is a joint first author and was actively involved in the study conception, design, strategic decisions and interpreting the findings, and helped draft the manuscript. SAC conceived the study, participated in its design, helped draft and revise the manuscript, made key strategic decisions and led the team. EA led the statistical and psychometric analyses. MOE guided, trained and performed the psychometric analyses and interpreted the findings. LP led the field work and training of interviewers for the study, participated in data analysis and item reduction and contributed to the manuscript. YWL participated in the study design and coordination and gave intellectual inputs on the manuscript. PMY was closely involved in the study design, data collection and field supervision. CBY led the data management component and quality control process and TYSJ assisted data collection, entry and analysis. CS steered the study design, concept and data analysis, interpreted the findings and helped draft the manuscript. All authors have read and approved the manuscript.
